# RETRACTION: CO_2_ Pneumoperitoneum Preserves *β*‐Arrestin 2 Content and Reduces High Mobility Group Box‐1 (HMGB‐1) Expression in an Animal Model of Peritonitis

**DOI:** 10.1155/omcl/9792742

**Published:** 2026-05-09

**Authors:** Oxidative Medicine and Cellular Longevity

RETRACTION: A. S. Montalto, A. Bitto, L. Minutoli, et al., “CO_2_ Pneumoperitoneum Preserves *β*‐Arrestin 2 Content and Reduces High Mobility Group Box‐1 (HMGB‐1) Expression in an Animal Model of Peritonitis,” *Oxidative Medicine and Cellular Longevity*, 2015, 160568, https://doi.org/10.1155/2015/160568.

The above article, published online on 24 February 2015 in Wiley Online Library (wileyonlinelibrary.com), has been retracted by agreement between the journal Editor‐in‐Chief, Jeannette Vasquez Vivar; and John Wiley & Sons Ltd. The retraction has been agreed due to multiple concerns raised with the figures on PubPeer [1]. Specifically:

• In Figure 2a, the ß‐actin bands appear similar to those presented in the:
  ◦ ß‐actin bands of Figure 2c
  ◦ ß‐actin bands in Figure 5c of [2]
  ◦ ß‐actin bands in Figure 2b of [3]
• In Figure 2b, the ß‐actin bands (lanes 1 and 2) appear similar to the ß‐actin bands (lanes 1 and 2) presented in Figure 4 of [4]
• In Figure 2b, the ß ‐actin bands appear similar to the ß‐actin bands (lanes 2‐4) shown in Figure 2a of [3]
• In Figure 3b, the HMGB‐1 bands appear similar to the BAX bands presented in Figure 6c of [3]

After assessment of the author’s response and the concerns raised by the editorial board, and considering the inability of the authors to provide the original Western blots, the results and conclusions presented can no longer be deemed reliable. The article is therefore retracted.

The authors did not respond when informed of the retraction.
